# Disrupted Brain Network Measures in Parkinson’s Disease Patients with Severe Hyposmia and Cognitively Normal Ability

**DOI:** 10.3390/brainsci14070685

**Published:** 2024-07-08

**Authors:** Karthik Siva, Palanisamy Ponnusamy, Malmathanraj Ramanathan

**Affiliations:** Department of Electronics and Communication Engineering, National Institute of Technology, Tiruchirappalli 620015, India; 408115053@nitt.edu (K.S.); palan@nitt.edu (P.P.)

**Keywords:** graph theory, feature ranking, nodal clustering coefficient, Parkinson’s Disease, hyposmia

## Abstract

Neuroscience has revolved around brain structural changes, functional activity, and connectivity alteration in Parkinson’s Disease (PD); however, how the network topology organization becomes altered is still unclear, specifically in Parkinson’s patients with severe hyposmia. In this study, we have examined the functional network topological alteration in patients affected by Parkinson’s Disease with normal cognitive ability (ODN), Parkinson’s Disease with severe hyposmia (ODP), and healthy controls (HCs) using resting-state functional magnetic resonance imaging (rsfMRI) data. We have analyzed brain topological organization using popular graph measures such as network segregation (clustering coefficient, modularity), network integration (participation coefficient, path length), small-worldness, efficiency, centrality, and assortativity. Then, we used a feature ranking approach based on the diagonal adaptation of neighborhood component analysis, aiming to determine a graph measure that is sensitive enough to distinguish between these three different groups. We noted significantly lower segregation and local efficiency and small-worldness in ODP compared to ODN and HCs. On the contrary, we did not find differences in network integration in ODP compared to ODN and HCs, which indicates that the brain network becomes fragmented in ODP. At the brain network level, a progressive increase in the DMN (Default Mode Network) was observed from healthy controls to ODN to ODP, and a continuous decrease in the cingulo-opercular network was observed from healthy controls to ODN to ODP. Further, the feature ranking approach has shown that the whole-brain clustering coefficient and small-worldness are sensitive measures to classify ODP vs. ODN, as well as HCs. Looking at the brain regional network segregation, we have found that the cerebellum and limbic, fronto-parietal, and occipital lobes have higher ODP reductions than ODN and HCs. Our results suggest network topological measures, specifically whole-brain segregation and small-worldness decreases. At the network level, an increase in DMN and a decrease in the cingulo-opercular network could be used as biomarkers to characterize ODN and ODP.

## 1. Introduction

A significant challenge in Parkinson’s Disease (PD) research is the validation of sensitive and reliable biomarkers for diagnosing various subtypes, monitoring clinical progression, and evaluating therapeutic interventions. The present-day clinical management of PD patients predominantly relies on clinical assessment and follow-up, with the highest diagnostic accuracy achieved through follow-up evaluations by trained PD specialists. Differentiating between Parkinson’s Diseases based solely on clinical assessment has proven unsatisfactory, particularly in the early stages after symptom onset. While MRI plays a crucial role, its primary function is to rule out other non-neurological conditions that might present with Parkinson-like symptoms. In the literature, imaging findings for PD conditions often need more specificity. Over the past three decades, brain imaging research has coalesced around delineating the brain’s resting networks, which is the pioneering approach to understanding the brain’s functions. This study originated from the hypothesis that the mathematical analysis of network connectivity using fMRI could potentially enhance the diagnostic sensitivity of imaging in Parkinson’s Disease and its subtype. The resting-state data presents a unique opportunity to comprehensively assess various networks, offering insights into the interplay between cognitive and motor networks in these diseases. The disease-specific connectivity patterns obtained from resting state MRI data could act as biomarkers for diagnosing and treating neurodegenerative diseases. The Parkinson-specific biomarkers might be helpful for the clinical diagnosis of patients with more complex symptoms or new symptoms, such as hyposmia, an early symptom of PD. An important biomarker for distinguishing PD patients and healthy subjects has been discovered as the resting state functional MRI connectivity [1]. However, its subgroup, such as PD with hyposmia symptoms, is still challenging. A few task-based fMRI and PET studies show significant findings relating to olfaction and PD [2,3,4]. Patients with PD showed significant activity reductions in their amygdala, hippocampus, and ventral striatum when exposed to odors in the scanner as compared to controls [2,5]. Structural and functional MRI research has shown brain abnormalities associated with cognitive deficits in PD patients [6]. Göttlich and colleagues [7], using graph theory analysis for fMRI data, have suggested that patients with Parkinson’s Disease exhibited poorer connections in the middle and medial orbitofrontal cortex compared to healthy controls. At the same time, Luo and colleagues [8] described the alteration in the brain’s topological organization during the early stage of drug-naïve patients with Parkinson’s Disease. Instead, using graph theory analysis, Fang and colleagues [9] reported no correlation between motor severity and topographic profiles in patients with Parkinson’s Disease. Moreover, Huang and colleagues’ study [10], using rsfMRI and Voxel-based Morphometry (VBM), suggested that graph theory can act as a potential analysis method in the diagnosis of Parkinson’s Disease and its subtypes. Using fMRI, Sako and colleagues [11] showed reduced functional connectivity between vermis X and cerebellar lobules IX without any evidence of compensatory effects in Parkinson’s Disease. Studies also have shown that severely hyposmic PD patients are more likely to develop or have cognitive and psychotic symptoms. Morley et al., in 2011, demonstrated the link between hyposmia and executive function deficits, memory deficits, and psychotic symptoms in PD patients. They suggest that hyposmia is pathognomonic for additional non-motor symptoms and that it might be used not only as a pre-diagnostic biomarker for PD but also as a marker of disease progression and the development of more severe non-motor symptoms [12]. Olfactory dysfunction is a failure of sensory and cognitive systems with significant links to other aspects of cognition. For example, MRI can be used to predict the likelihood of PD patients having moderate cognitive deficits or dementia. In this study, we assessed the network topology using various graph theory measures aiming to provide a biomarker that is helpful for an early diagnosis of brain abnormalities in PD patients. The novelty of this study is the use of various graph theory measures, including “network integration, segregation, efficiency, and small-worldness” with a feature ranking approach, which not only detects abnormalities in Parkinson’s with hyposmia and cognitively normal Parkinson’s patients also provides which measure could be sensitive to use as a biomarker for diagnosis and targeted treatment.

The highlights of the proposed work are

1. Graph theory, an efficient mathematical technique, is used to demonstrate brain alteration in Parkinson’s Disease and its subtypes.
2. At the whole-brain level, we have found a significant decrease in the clustering coefficient (segregation) in Parkinson’s with hyposmia patients compared to Parkinson’s with normal cognitive ability.
3. At the brain network level, we found a progressive increase in the Default Mode Network (DMN) from healthy controls to ODN to ODP as a compensatory mechanism of brain networks.
4. A continuous decrease in the cingulo-opercular network was observed in both Parkinson’s groups when compared to the healthy controls.
5. The above results could be considered biomarkers or pre-diagnostic tools to understand Parkinson’s Disease for the next level of therapeutic treatment.

In this paper, Section 1 explains our objective and the literature, Section 2 explains our workflow and graph theory properties, Section 3 explains our obtained results from this methodology, Section 4 explains the interpretation of our results with previous literature, and Section 5 highlights the contributions of our work.

## 2. Materials and Methods

### 2.1. Materials and Methods

**Figure 1:**
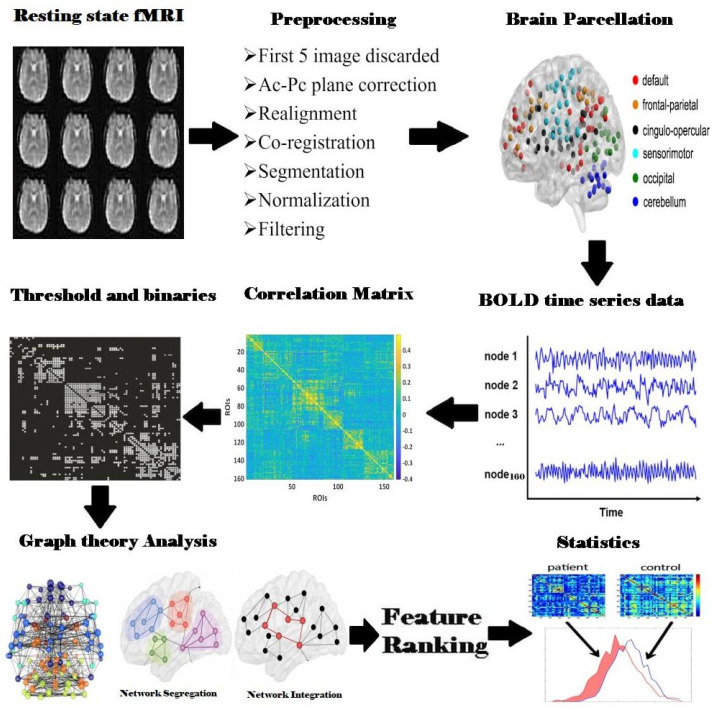
Flowchart illustrating the complete steps for resting-state fMRI (rsfMRI) data analysis used in constructing functional brain networks and performing graph theory analysis in the human brain. The steps include rsfMRI data collection, preprocessing, brain parcellation using Dosenbach’s template, BOLD time series extraction by averaging signals within each region of interest (ROI), constructing correlation matrices for each subject, thresholding and binarizing these matrices, performing graph theory analysis, feature ranking, and statistical analysis.

This MRI data was obtained from the OpenfMRI database, and the accession number is ds000245 [13]. The dataset included the structural and functional data of 45 participants. It provided each participant’s age information, ACE-R (Addenbrooke’s Cognitive Examination Revised) scores [14], OSIT-J (Odor Stick Identification Test for the Japanese) scores [15], Mini-Mental State Exam (MMSE) scores, and the disease duration of each PD participant as well as age at diagnosis. The OSIT-J comprises 12 odors familiar to Japanese people and is a widely used tool for assessing olfactory function in Parkinson’s Disease (PD) patients. The ACE-R evaluates six cognitive domains: orientation, attention, memory, verbal fluency, language, and visuospatial ability. It is utilized to diagnose dementia in PD patients. In this study, the definition of “Parkinson’s patient with severe olfactory loss” is explained as those with an OSIT-J score of 4 or less. This threshold indicates significant olfactory impairment. Additionally, to ensure that severe cognitive impairment did not confound our results, we excluded patients with ACE-R scores of 88 or below, corresponding to cognitive impairment. For detailed demographic information, please refer to the original article by Yoneyama et al., 2018 [13]. Among the total 45 subjects, fifteen healthy individual subjects (63.3 ± 5.2 years; 8 females), fifteen cognitively normal Parkinson’s Disease patients (64.4 ± 7.2 years; 9 females), and fifteen Parkinson’s Disease patients with severe hyposmia (70.7 ± 4.8 years; 8 females) were chosen. The disease duration for ODP (5.9 ± 3.7 years) and ODN (6.1 ± 3.2 years) showed no significant differences. The ACE-R scores were 94.3 ± 3.4 for ODP, 96.1 ± 3.1 for ODN, and 97.3 ± 2.9 for HCs, with no significant differences. The MMSE scores were 29.1 ± 1.1 for ODP, 29.0 ± 1.3 for ODN, and 29.5 ± 0.6 for HCs, showing no significant differences. OSIT-J scores, indicative of olfactory function, were significantly lower in the ODP group (1.7 ± 1.1) compared to the ODN (7.5 ± 1.5) and HC (10.4 ± 1.3) groups (*p* < 0.0001). Based on the scores from a smell recognition test (OSIM-J), hyposmia was assessed. The 3T rsfMRIs were acquired (echo-planar images (EPI) sequence with 2500 ms repetition time (TR), 3 mm of slice thickness, 30 ms echo time (TE), 64 × 64 matrix dimension and FOV = 192 mm). For anatomical reference, T1-weighted images were acquired (2500 ms repetition time, 2480 ms echo time, 3-mm slices thickness, 64 × 64 matrix dimension). The potential confounders, such as age and gender, were controlled by employing Dukart et al.’s (2012) methodology [16]. A regression model for age and gender was generated at each voxel using data from the healthy control (HC) group, and the estimated regression coefficients were used to regress age and gender effects from the data of both the HC and patient groups. This procedure ensures that age or gender differences between groups do not confound the results. Additionally, other potential confounders were controlled for in our study. The mean disease duration for the ODP (5.9 ± 3.7 years) and ODN (6.1 ± 3.2 years) groups was not significantly different, ensuring that disease duration did not confound the results. Cognitive assessments using the Addenbrooke’s Cognitive Examination Revised (ACE-R) and Mini-Mental State Examination (MMSE) ensured that cognitive differences did not influence the rsfMRI results. By implementing fMRI pre-processing steps (Figure 1) and controls, our study aimed to minimize the impact of confounding variables, thereby providing more accurate and reliable results regarding the functional connectivity changes in Parkinson’s Disease.

### 2.2. fMRI Data Pre-Processing

The SPM8 (statistical parametric mapping) was used to preprocess the fMRI data. The T1-MPRAGE and fMRI data were adjusted to the AC-PC plane using SPM 8, followed by the reorientation of the T1-MPRAGE with fMRI images. The preprocessing of fMRI data comprises four stages: realignment, co-registration, segmentation, and normalization. The main objective of performing realignment is to remove the movement artifacts present in the fMRI time series data. The co-registration phase of pre-processing was executed further to ensure that the functional and structural MRI images were in the same plane. The normalization stage was executed based on the T1 segmentation tissue probability maps of white matter (WM) and cerebrospinal fluid (CSF). This stage warps the size of the image in order to fit into a standardized template with average dimensions using the third-degree interpolation method to ensure accurate registration into a different space.

### 2.3. Construction of Brain Network

We used the Dosenbach-160 template to parcellate the functionally segregated brain regions of interest (ROIs). These segregated parts cover the whole cortical, subcortical, and cerebellum regions for both hemispheres [17]. The nodes of functional networks were transformed from the unsmoothed normalized rsfMRI data into 160 spheres of the Dos-160 template with a radius of 5 mm, using the MarsBaR 0.43 toolbox. The time series of every ROI was computed by taking an average of all the voxels present in each corresponding ROI. Followed by the functional connectivity matrix generated using Pearson’s correlation between all the rsfMRI time series, the resultant correlation matrix has a size of 160 × 160 [18].

### 2.4. Graph Theory Analysis

We analyzed brain topological organization using a graph theory approach. In this regard, popular graph measures such as network segregation (clustering coefficient (CC), modularity), network integration (participation coefficient, path length (PL)), small-worldness (SW), efficiency (local efficiency (LE), global efficiency (GE)), degree, between centrality, and assortativity were used. In studying whole-brain networks, key metrics often employed to describe global organization are the “clustering coefficient” and “average path length”. The average clustering coefficient quantifies the level of segregation within a network by measuring the connection probability of the nearest neighboring nodes, thereby reflecting local connectedness. In contrast, the average path length, based on all possible pairs of nodes in a network, measures global connectedness by counting the number of edges traversed from one node to another. This metric is inversely related to network integration, with a shorter average path length indicating better integration. These two metrics are crucial for distinguishing between three fundamental network configurations: regular, small-world, and random. A regular network, characterized by a high clustering coefficient, has many local connections but few distant ones, resulting in a high average path length. Conversely, a random network features limited local connections and numerous distant connections. The small-world network, which merges the benefits of regular and random networks, is considered the most efficient topology due to its good local and global connectedness.

#### Network Topology Analysis

To compute the graph measures, we used functional connectivity matrices which were generated using Pearson’s correlation. The properties of the functional networks were considered as N × N (i.e., 160 × 160) by the undirected graph of a binary network, G(N,E), in which G denotes the non-zero subset. G consists of edges denoted by E that are coefficients obtained by intermodal correlation (i.e., Fisher’s Z values) and nodes (N—ROIs) as a number of operational links/connectivity amongst N number of nodes [19]. The proposed study employs an undirected binary network graph to compute the properties, enabling the comparison of nodal characteristics across participants and groups. A sparsity threshold was utilized to ensure that the same number of network edges were present for each participant, thereby retaining only the connections with edge strengths exceeding the predefined threshold [20]. A sparsity threshold (S) was used in the sparsity range from 0.1 (10%) to 0.5 (50%) sparsity thresholds (with a tolerance of 0.05) for the purpose of preventing excessive fragmentation of the network at sparser thresholds [18].

Clustering Coefficient (CC): Clustering coefficients calculate triangles around a node and its neighbors and are a measure of network segregation. Clustering coefficients can be used to quantify network interconnection and how well networks separate themselves. In order to calculate coefficients, the method described by Watts et al., 1998 [21] was applied, which requires symmetrical weights between node “*i*” and the other nodes “*j*”. *C_i_* is the ratio of all attainable connections between the nodes in a neighborhood divided by the number of existing connections. In a network, the clustering coefficient is determined by the average of the absolute clustering coefficients [22]. *CC* can be mathematically described as follows:

(1) $$CC=\frac{1}{n}\sum_{i\in N}C_{i},$$

where $C_{i}=\frac{2E_{i}}{Q_{i}(Q_{i}-1)}$, *N* is the total number of nodes, *E_i_* is the number of existing connections of node *i* and *Q_i_* is the degree of node *i*. The degree indicates how much connectivity the node has with the rest of the network. Those nodes with a higher degree have more connections to each other. Nodes with higher *C_i_* values are hub regions.

Path Length (PL): There are paths between two nodes in a network that indicate a series of unique edges. An average shortest path connecting two nodes of a network is called the characteristic path length of a network “*L*”. Network integration is measured by “*L*”, which indicates how well the elements of the network are connected. Based on the shortest path length, the algorithm finds the path with the lowest cost/edges from any given node to any other node. To determine the shortest path length between node “*L_i_*” and all other nodes, we use the following formula.

(2) $$L_{i}=\frac{1}{N(N-1)}\sum_{i\in G}\sum_{i\neq j\in N}min\{L_{i,j}\}$$

where *min*{*L_i_*_,*j*_} is the shortest absolute path length between the nodes “*i*” and “*j*”. In the network “*L*”, the shortest path length is defined as the sum of the shortest absolute path lengths [23], according to the following formula:

(3) $$L=\frac{1}{N}\sum_{i\in G}L_{i}$$

Small-Worldness (SW): According to the following criteria, a network is considered small-world if it meets both conditions: $\gamma =\frac{C_{i}}{C_{Rand}}>1$ and $\lambda =\frac{L_{i}}{L_{Rand}}\approx 1.$

*C_i_* is the absolute clustering coefficient and *L_i_* is the absolute path length, where *C_Rand_* and *L_Rand_* are the mean clustering coefficient and characteristic path length of the matched random networks [21]. Networks can be considered small-world structures (towards regular properties) if they satisfy $\sigma =\frac{\gamma }{\lambda }>1$.

Network Efficiency (EF): There are two types of network efficiency: (1) the global efficiency, which measures integration across the entire network; and (2) the local efficiency, which measures separation over all nodes. We computed the global efficiency “$E_{g}$” based on the average inverse shortest path length in the network. A topologically integrated network has a high “$E_{g}$” value.

(4) $$E_{g}=\frac{1}{N(N-1)}\sum_{i\neq j\in L_{i,j}}{L_{i,j}}^{-1}$$

The local efficiency of node *i* $E_{loc}$ is determined by the average inverse shortest path length defined in the subgraph that contains *i* and its neighbors.

(5) $$E_{loc}=\frac{1}{N}\sum_{i\in G}E\left(G_{i}\right)$$

$E_{loc}$ is used to indicate how fault-tolerant the whole network is, thus it represents how efficient it is to communicate between the first neighbors of node “*i*” when “*i*” is removed [23].

Betweenness Centrality: The fraction of all shortest connections in the network that contain a certain node is referred to as the betweenness centrality. A large number of shortest paths pass through nodes with high values of betweenness centrality [24,25]. The betweenness centrality for node *i* is calculated as follows:

(6) $$b_{i}=\frac{1}{\left(n-1\right)\left(n-2\right)}\sum_{h,j\in N}\frac{\rho_{hj}\left(i\right)}{\rho_{hj}},$$

where $\rho_{hj}$ represents the number of shortest paths between *h* and *j*, while $\rho_{hj}$(*i*) represents the number of shortest paths between *h* and *j* that cross *i*.

Modularity: Quantifying the modularity of a network *Q* is a method of defining how clearly it can be divided into groups [24,25]:

(7) $$Q=\frac{1}{l}\sum_{i,j\in N}\left(a_{ij}-\frac{k_{i}k_{j}}{l}\right)\delta_{m_{i,}m_{j}},$$

A module *m_i_* contains a node *i*, and $\delta_{m_{i,}m_{j}}$ = 1 if $m_{i}=m_{j}$, and otherwise 0.

This metric quantifies the degree to which a graph can be partitioned into distinct communities or modules. The calculation of modularity relies on a pre-defined community structure.

Participation Coefficient: By measuring the participation coefficient, it is possible to view the connectivity diversity within individual modules. The Louvain algorithm was used to determine the community allocation of individual modules with a resolution parameter equal to one. By maximizing within-group edges and minimizing between-group edges, one strives to form a subdivision of the network [24,25]. The participation coefficient was calculated by:

(8) $$y_{i}=1-\sum_{m\in M}{\left(\frac{k_{i}(m)}{k_{i}}\right)}^{2},$$

where $k_{i}(m)$ is determined by the degree of connection between node *i* and nodes in that module *m*.

Assortativity: Whenever a node with a certain degree is linked to another node with a similar degree, that is called assortativity. It is determined by correlating the degree of each node with the mean degree of its neighbors [26].

(9) $$r=\frac{l^{-1\;\;\;}\sum_{i,j\in L}k_{i\;\;\;}k_{j}-{\left[l^{-1}\sum_{i,j\in L}\frac{1}{2}(k_{i}+k_{j})\right]}^{2}}{l^{-1}\;\sum_{i,j\in L}\frac{1}{2}\left(k_{i}^{2}+k_{j}^{2}\right)-{\left[l^{-1}\sum_{i,j\in L}\frac{1}{2}(k_{i}+k_{j})\right]}^{2}},$$

where $k_{i}$ and $k_{j}$ are the respective degrees of the nodes *i* and *j*, and *l* is the number of edges in the graph. In directed and weighted networks, the corresponding assortativity coefficients are calculated using the weighted and directed variants of degree or strength. A positive assortativity coefficient signifies that nodes tend to connect to other nodes with similar degrees or strengths.

### 2.5. Features Ranking

Using a feature ranking approach based on neighborhood component analysis (NCA), we determined the sensitive graph measure to distinguish these three different groups. It involves using non-parametric analysis to determine which data features have been used to improve prediction accuracy in classification algorithms. In this framework, feature weights were learned to minimize an objective function that measures the average leave-one-out classification loss over the input data. An NCA analysis was performed using the MATLAB 2021a function fscnca [27].

(10) w=argmin1/n∑i=1n∑j=1,j≠inpij  l(yi, yj)+β∑r=1pwr2 ,

(11) $$l\left(y_{i},y_{j}\right)=\left\{\begin{array}{c} 1,\;\;if\;\;y_{i}\neq y_{j}\\ 0,\;\;otherwise \end{array}\right.\;,$$

where *w* represents the feature weights; *i* and *j* are indices for different subjects’ brain network data; $y_{i}$ and $y_{j}$ are the class labels indicating whether a subject belongs to the healthy control group, Parkinson’s with normal cognition group, or Parkinson’s with severe hyposmia group; and *p_ij_* represents the probability that the brain network of subject *j* is the nearest neighbor to the brain network of subject *i*. One of the primary advantages of NCA over other feature selection methods, such as Principal Component Analysis (PCA) or Recursive Feature Elimination (RFE), is its direct optimization for classification performance rather than relying on intermediate steps like feature variance or linear combinations of features. Unlike PCA, which reduces dimensionality by projecting data into a new space, NCA retains the original feature space, making the results more interpretable. Additionally, NCA is less prone to overfitting compared to RFE because it evaluates feature importance in a leave-one-out cross-validation framework, providing a more robust selection of features.

### 2.6. Statistical Analysis

An important issue in fMRI data analysis is the specification of an appropriate threshold for statistical analysis. Statistical analysis in fMRI studies includes large-scale multiple comparisons and the application of individual tests from one time series to another time series. Methodologically, it was implemented by initially estimating the statistical difference between the groups at each sparsity level to check for overall group differences in graph theory properties. Since the comparison was between any two groups with that of controls (e.g., ODN, ODP), FDR correction for the three different level group comparisons was tested. The data were analyzed using MATLAB. There were some brain regions that had significant group differences between the group comparisons. The negative t-value indicated decreased clustering coefficients in one group compared to another. The graph theory measures were examined with the range of 0.1 (10%) to 0.5 (50%) sparsity thresholds in all nodes for each patient and healthy control, and two-sample two-tailed t-tests were used to calculate the group differences in MATLAB. Multiple comparisons were corrected using 5% confidence intervals of the false discovery rate (FDR < 0.05). Brain regions’ significant changes were visualized using BrainNet Viewer 1.43 [28].

This study has several limitations. Firstly, we utilized image data from a public database, where the quality control of the dataset may not be optimal. Additionally, each group in our study consisted of only 15 subjects, which is inadequate for robust classification analyses. Future research will focus on applying dynamic communicability analysis, an advanced graph theory analysis technique, to provide deeper insights into brain function and dysfunction in Parkinson’s Disease with hyposmia patients.

## 3. Results

### 3.1. Brain Functional Connectivity

**Figure 2:**
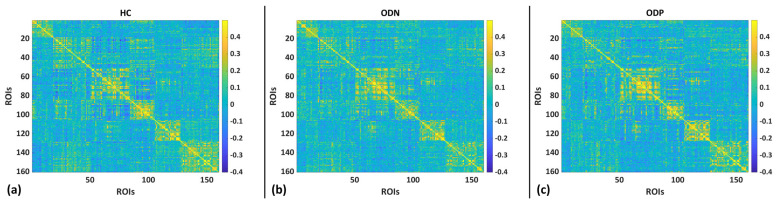
Brain functional connectivity for (**a**) healthy controls (HCs), (**b**) Parkinson’s with normal cognitive ability (ODN), and (**c**) Parkinson’s with severe hyposmia (ODP).

To understand the brain functional network topological alteration in Parkinson’s with severe hyposmia (ODP) and Parkinson’s with normal cognitive ability (ODN), we first analyzed the brain functional connectivity (FC) differences. We noted that ODP patients have relatively lower connectivity compared to ODN and healthy controls (HCs) (Figure 2). However, these differences did not survive statistical thresholds.

### 3.2. Brain Functional Network Topology

**Figure 3:**
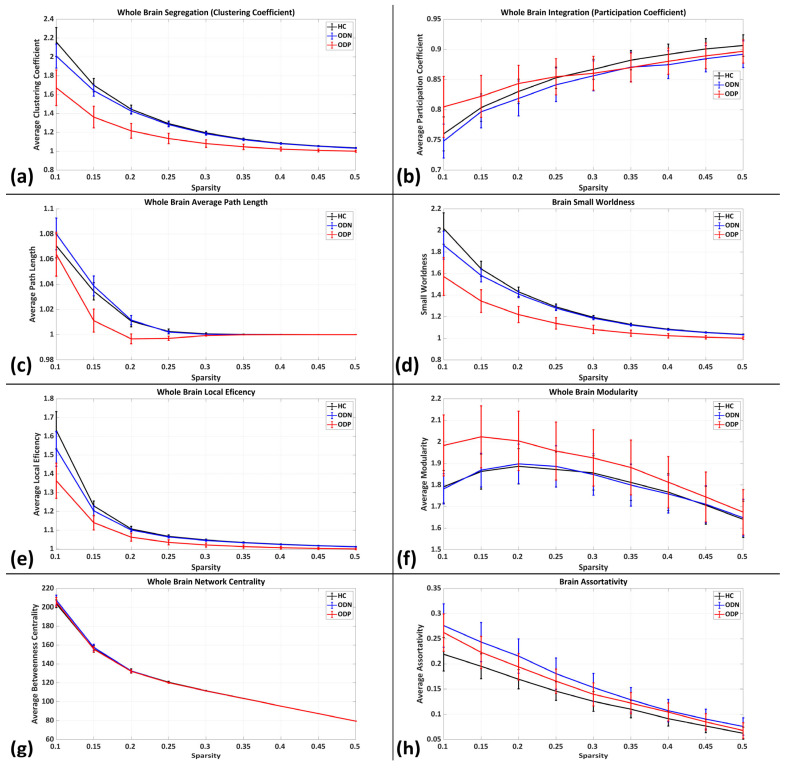
Brain functional network topology (graph measures) of healthy controls (HCs) (black), Parkinson’s with normal cognitive ability (ODN) (blue), and Parkinson’s with severe hyposmia (ODP) (red) for (**a**) whole-brain segregation (clustering coefficient), (**b**) whole-brain integration (participation coefficient), (**c**) whole-brain average path length, (**d**) small-worldness, (**e**) whole-brain local efficiency, (**f**) whole-brain modularity, (**g**) whole-brain network centrality, and (**h**) assortativity.

**Figure 4:**
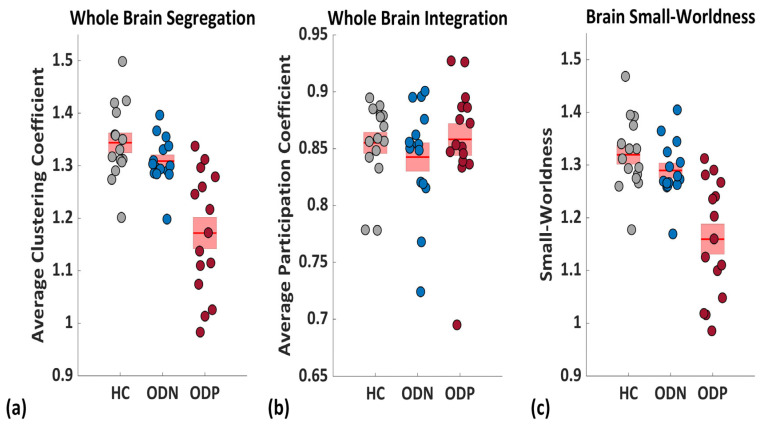
Bar graph of (**a**) whole-brain segregation, (**b**) integration, and (**c**) small-worldness for healthy controls (HCs) (gray dots), Parkinson’s with normal cognitive ability (ODN) (blue dots), and Parkinson’s with severe hyposmia (ODP) (Red dots).

To understand the altered functional connectivity underlying structures in ODP vs. ODN, we have assessed network topological alteration in the aspect of brain network integration, segregation, efficiency, randomness (i.e., small-worldness), and network density properties (Figure 3 and Figure 4). We have noted in ODP significantly lower segregation (i.e., whole-brain clustering coefficients), local efficiency, and small-worldness compared to ODN and HCs. On the other hand, we have not found differences in network integration in ODP as compared to ODN and HCs, as well as ODN compared to HCs, which indicates that the brain network becomes fragmented in ODP and does not have significant interlobular interactions (Figure 3 and Figure 4). We also have not found any differences in other graph measures including path length, modularity, network centrality, assortativity, and strengths. ODN has shown relatively lower brain segregation (i.e., whole-brain clustering coefficients) as compared to HCs. We also have not found any differences in other graph measures between ODN and HCs (Figure 3).

### 3.3. Findings from the Networks of Brain

**Figure 5:**
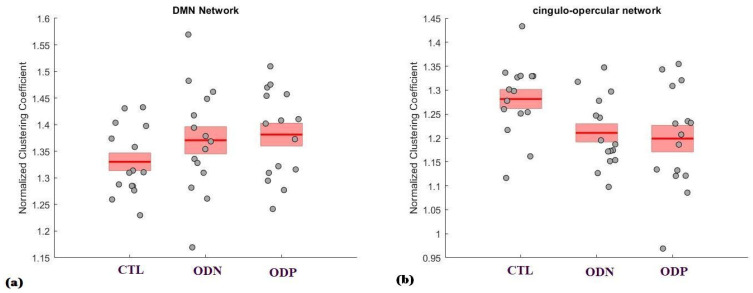
The figure presents a comparison of the normalized clustering coefficient across three groups, healthy controls (CTL), Parkinson’s patients with normal cognitive ability (ODN), and Parkinson’s patients with severe hyposmia (ODP), within two specific brain networks: (**a**) Default Mode Network (DMN) and (**b**) cingulo-opercular network.

In Figure 5a, DMN was found to be highest in the ODP group, followed by the ODN group, and lowest in the CTL group. This trend indicates that Parkinson’s Disease patients with hyposmia exhibit greater DMN connectivity as compared to those with cognitive normality, and both groups of Parkinson’s patients exhibit higher connectivity than healthy controls. In Figure 5b, the ODN group is lower than the CTL group, indicating a decrease in network connectivity associated with cognitively normal Parkinson’s Disease. Furthermore, the ODP group exhibited even lower mean connectivity than the ODN group, suggesting that the presence of hyposmia in Parkinson’s Disease patients is associated with a further reduction in cingulo-opercular network connectivity. This indicates potential network-specific variations in brain connectivity associated with the three groups.

### 3.4. Brain Regional Alteration

**Figure 6:**
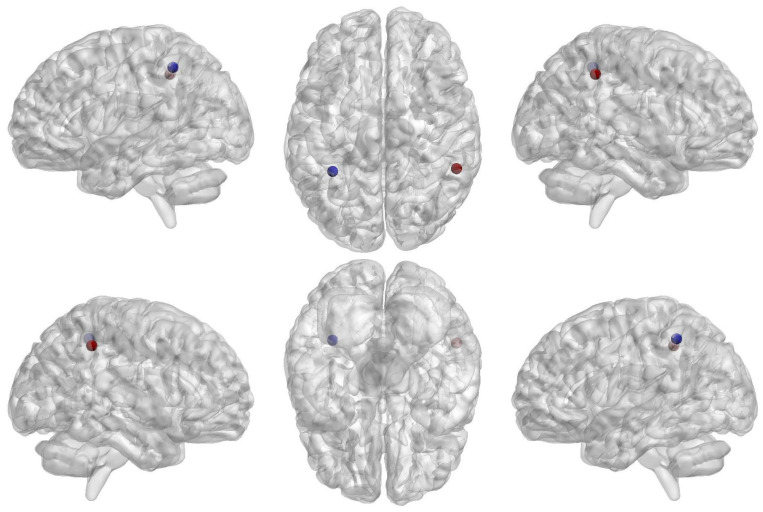
Glass brain view of group differences for brain regional differences between healthy controls (HCs) and Parkinson’s with normal cognitive ability (ODN), which was computed using nodal network segregation (i.e., nodal clustering coefficient). The dotted brain regions showed decreased network segregation in ODN compared to HCs with *p*  <  0.005. The size and color (blue to red jet color) indicate the “t-values” of statistical differences between ODN and HCs.

Further, we have looked at the regional changes using nodal clustering coefficients as whole-brain clustering coefficients show significant differences between groups and feature ranking shows the highest weight to classify the groups. In ODN as compared to HCs, we noted only left and right parietal areas with *p* < 0.005 (Figure 6 and Table 1).

**Figure 7:**
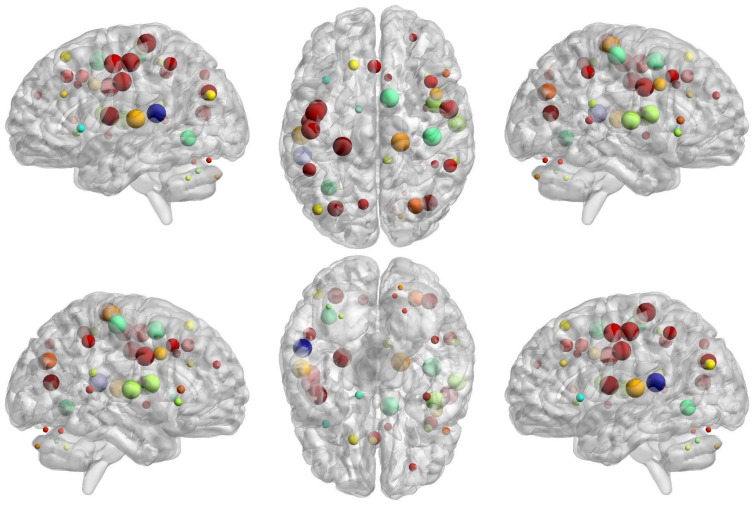
Glass brain view of group differences for brain regional differences between healthy controls (HCs) and Parkinson’s with severe hyposmia (ODP), which was computed using nodal network segregation (i.e., nodal clustering coefficient). The dotted brain regions show decreased network segregation in ODP compared to HCs with a multiple comparisons correction of FDR  <  0.05 for no of ROIs (N  =  160). The size and color (blue to red jet color) indicate the “t-values” of statistical differences between ODP and HCs.

**Figure 8:**
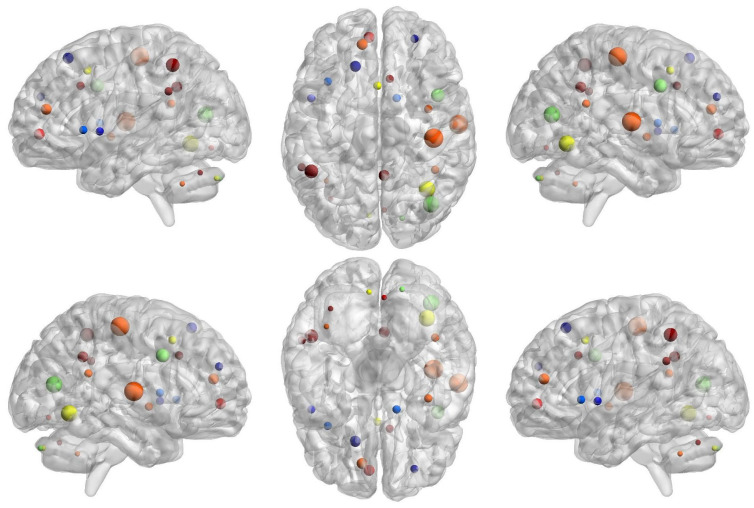
Glass brain view of group differences for brain regional differences between Parkinson’s with normal cognitive ability (ODN) and Parkinson’s with severe hyposmia (ODP), which was computed using nodal network segregation (i.e., nodal clustering coefficient). The dotted brain regions show decreased network segregation in ODP compared to ODN with a multiple comparisons correction of FDR  <  0.05 for no of ROIs (N  =  160). The size and color (blue to red jet color) indicate the “t-values” of statistical differences between ODP and HCs.

In ODP, we have found a severe reduction in local network segregation compared to ODN and HCs (Figure 7 and Figure 8). The affected brain regions are in the cerebellum, fronto-parietal, and sensorimotor areas (Figure 7 and Figure 8, Table 1). Specifically, in ODN as compared to ODP, we have noted decreased network segregation at the bilateral inferior cerebellum, right medial cerebellum, bilateral insula, left ventromedial prefrontal cortex (vmPFC), ventral frontal cortex (VFC), superior frontal, middle frontal cortex (mFC), right anterior prefrontal cortex (aPFC), dorsal frontal cortex (dFC), dorsal ACC, right basal ganglia, bilateral parietal, precuneus, right sup temporal, and occipital areas (Figure 8, Table 1).

In ODP as compared to HCs, these areas are further found to be more impacted (i.e., decreased) in network segregation in addition to the regions of the left thalamus, ACC, right vFC, pre-SMA, left precentral gyrus, bilateral IPL, temporal, and left occipital (Figure 7, Table 1).

### 3.5. Feature Ranking

**Figure 9:**
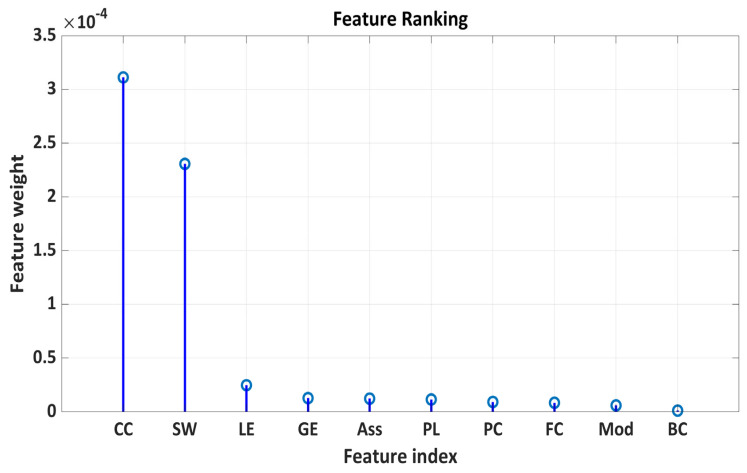
Feature ranking of functional connectivity and graph measures to classify healthy controls (HCs), Parkinson’s with normal cognitive ability (ODN), and Parkinson’s with severe hyposmia (ODP). CC = whole-brain clustering coefficient (CC); SW = small-worldness; LE = whole-brain local efficiency; PL = whole-brain average path length; GE = whole-brain global efficiency; Ass = whole-brain assortativity; PC = whole-brain participation coefficient; FC = whole-brain average functional connectivity; Mod = whole-brain modularity; BC = whole-brain network centrality.

To determine which graph measure is sensitive to distinguish between ODP, ODN, and HCs, we have used a feature ranking approach using feature weights by diagonal adaptation of neighborhood component analysis (NCA). We have found that the whole-brain clustering coefficient (CC) and small-worldness (SW) are sensitive measures to classify ODP vs. ODN, as well as HCs (Figure 9).

## 4. Discussion

### 4.1. Whole-Brain Segregation Decrease

Functional connectivity and network topology have emerged as potential approaches to explain the functional properties of the brain and its alteration in neurological deficits. Previous studies showed reduced functional connectivity and an alteration in network topological organization in patients with Parkinson’s; however, the neural dynamics in Parkinson’s with severe hyposmia (ODP) are still unclear. Using a graph theory-based approach, this study has shown that ODP had decreased functional connectivity compared to ODN and HCs. Diving deeper, we have demonstrated decreased connectivity due to altered network topological organization. The ODP brain dynamics had lower network segregation, local efficiency, and small-worldness. This pattern has also been prominent in ODN as compared to healthy controls. Diving into regional brain alterations, ODP has shown significantly lower network segregation (clustering coefficient) than ODN and HCs in limbic areas and frontal areas, including the ventral prefrontal cortex, parietal, temporal, occipital areas, and the cerebellum. However, our study has noted bilateral parietal network segregation differences in ODN compared to ODP. Our finding of decreased connectivity [7,29,30,31,32] and disrupted network organization in Parkinson’s Disease is in line with previous studies [8,9,11,29,33]. In Parkinson’s Disease, the network segregation measured by the clustering coefficient was decreased [7,8,9,33]. Further decreased local efficiency and small-worldness in Parkinson’s Disease have been reported by Luo et al. and Sreenivasan et al. [8,33]. Here, using a novel feature ranking algorithm, we have shown that across various graph theory measures, network segregation (i.e., clustering coefficient), small-worldness, and local efficiency are the key measures to distinguish not only Parkinson’s Disease from healthy controls but also to distinguish Parkinson’s with severe hyposmia patients from cognitively normal Parkinson’s patients. Parkinson’s with severe hyposmia showed severe damage of network segregation at the whole-brain and regional level and reduced brain small-worldness, suggesting decreased connectivity in Parkinson’s with severe hyposmia [13] due to network fragmentation and inefficient network organization.

Recent studies have shown olfactory dysfunction in patients with Parkinson’s Disease [34,35,36]. Although different hypotheses have been proposed, the potential causes of olfactory dysfunction are still unknown. It has been hypothesized that Parkinson’s Disease starts in autonomic neurons, olfactory bulbs, and the anterior olfactory nucleus [37,38]. Further, Iannilli et al. have shown decreased EEG global field power in fronto-parietal and central electrodes and suggested olfactory dysfunction in patients with Parkinson’s related to peripheral and central nervous system changes [39]. Wang et al. have shown decreased grey and white matter functional covariance in olfactory-related brain regions [40], which is in line with our findings. We suggest that decreased connectivity and EEG global power in severely hyposmic Parkinson’s could be due to a reduction in brain network segregation and inefficient network reorganization, specifically at the limbic and frontal areas (ventral middle prefrontal cortex), inferior parietal and occipital regions, and the cerebellum. Our findings suggest complex network dysfunction and inefficient network reorganization that exceeds structural and functional dynamics, observed in the olfactory bulb and mesolimbic cortices, and thus demonstrates a vital contribution to the degenerating brain. These areas’ network segregation could be used to classify ODP and ODN and assess the prognosis and effect of targeted treatment. For example, repeated transcranial magnetic stimulation (rTMS) or transcranial direct current stimulation (tDCS) can be used, and network segregation and efficiency can be assessed to understand individual patients’ responses to the targeted treatment.

### 4.2. Comparison with Yoneyama et al., 2018 [13]

Yoneyama et al. 2018 assessed PD patients with mild or no hyposmia and PD patients with severe hyposmia along with age-matched healthy subjects and showed that PD with hyposmia patients have severely diminished functional connectivity in various functional modules including the amygdala and inferior parietal lobule, lingual gyrus, and fusiform gyrus, which have been associated with cognitive performance [13]. A cortical gray matter volume analysis with seed-based correlation connectivity analysis and a whole-brain canonical resting-state fMRI connectivity analysis using ICA have been used by Yoneyama et al., 2018 [13]. The study shows alterations in connectivity between the three nuclei of the amygdala “seeds” and many other brain areas. The difference in decreased and increased functional connectivity has been observed between the PD patients with severe hyposmia and the healthy control group [13]. A seed can be chosen based on prior data on how the brain region is significantly affected by PD; for example, a region known to atrophy as a direct consequence of PD. Their data-driven ICA analysis discovers changes in brain connectivity within the precuneus network and visual networks in the PD with severe hyposmia [13]. The Independent Component Analysis approach has a limitation for the number of independent components chosen, and the chosen independent components may lead to subject-specific networks being missed or overlooked. Importantly the above study on functional connectivity and independent component analysis did not address how severely decreased activation and connection alter the network topological organization, which could be interesting in providing fundamental insights into the functional alteration of brain dynamics in patients with Parkinson’s.

### 4.3. Olfactory Literature Comparition

Our results showed that the networks of both ODP and ODN patient groups exhibited decreased nodal clustering coefficients in the anterior prefrontal cortex (aPFC) region of the brain. They have also suggested decreased connections in patients with Parkinson’s Disease. Moreover, the study results are in line with the data reported by Luo et al. (2015) [8] that illustrated damage in the organization of brain topology in early-stage drug-naïve patients with Parkinson’s Disease. In particular, previous studies [7,8,41] have reported a decrease in connections in the middle and medial orbitofrontal cortex compared to age-matched healthy controls. Many previous studies evaluated olfactory dysfunction in patients with Parkinson’s Disease [34,35,36]. The potential causes of olfactory dysfunction are still to be narrowed down, although different hypotheses have been proposed. In particular, the Braak model hypothesized that Lewy pathology in patients with Parkinson’s Disease starts in autonomic neurons, olfactory bulbs, and the anterior olfactory nucleus [37,38,42,43]. Previous neuroimaging studies have reported that the olfactory dysfunction could be related to the olfactory bulb volume [44,45] or to the piriform cortex [46] and the orbitofrontal cortex [47,48]. Consequently, the causes of olfactory dysfunction in patients with Parkinson’s Disease are not totally understood but probably are related to peripheral and central nervous system changes; specifically, few studies have reported rich connectivity in the angular gyrus region [39]. Monchi et al.’s study [49] proposes that in patients with Parkinson’s Disease, the nigrostriatal dopamine depletion exhibited decreased cortical connectivity. Moreover, a previous study has reported visuospatial dysfunction and hyposmia in patients with Parkinson’s Disease [50].

Betweenness centrality connectivity changes in the olfactory regions, as provided in Supplementary Figure S1, highlight the progressive decline in betweenness centrality within the olfactory network regions of interest (ROIs) from healthy controls (HCs) to ODN and further to those with olfactory dysfunction (ODP). This suggests a localized fragmentation of the network, which becomes more significant as olfactory function diminishes in Parkinson’s Disease patients. This localized disruption might have been counteracted by compensatory mechanisms, such as increased activity in other brain regions or the reorganization of existing connections, maintaining overall network integration [51,52]. Additionally, the progressive increase in the Default Mode Network (DMN) from HCs to ODN to ODP supports the hypothesis that the DMN plays a critical role in preserving global network integration despite localized disruptions.

### 4.4. Cognitive Impairment

Metabolic imaging using spatial covariance analysis has advanced our understanding of the network-related functional changes linked to cognitive symptoms in Parkinson’s Disease (PD). A study by Huang et al. (2007) applied regression analysis alongside principal component analysis (PCA) to pinpoint a distinct metabolic pattern associated with executive function in non-demented PD patients [53]. This PD-related cognitive pattern (PDCP) features metabolic decreases in the pre-SMA, prefrontal cortex, precuneus, and parietal association areas, accompanied by increases in the cerebellar vermis and dentate nuclei. In non-demented PD patients, PDCP expression has been linked to performance on executive function tests such as the California Verbal Learning Test, Trail Making Test B, and the Stroop Color Test. A cross-sectional study of network values in over 100 subjects demonstrated a significant correlation between elevated PDCP and cognitive impairment, spanning from patients with intact cognition to those with dementia [54,55,56].

The significant reductions in network segregation, local efficiency, and small-worldness observed in Parkinson’s Disease patients with severe olfactory hyposmia suggest disruptions in brain network organization, impacting cognitive functioning and daily life. These changes imply less distinct and efficient information transfer within specialized networks, leading to deficits in attention, executive functions, and memory [57,58]. Additionally, reducing the cingulo-opercular network, which is crucial for cognitive control and attention, further contributes to these impairments [59]. The observed increase in the Default Mode Network (DMN) might reflect a compensatory mechanism, but excessive DMN activity can also impair attention and increase distractions [60].

### 4.5. DMN and Cingulo Opercular Decrease

Jin et al. (2011) have highlighted high regional efficiency in the supplementary motor cortex (SMA) in Parkinson’s Disease, which underscores the compensatory mechanisms the brain may employ in response to motor deficits [61]. Additionally, Solodkin et al. (2011) have identified progressive organizational changes in the lateral cerebellum and thalamus, which are critical regions involved in motor and cognitive functions. This suggests that these regions may undergo significant structural and functional reorganization as the disease progresses [62]. Furthermore, Fang et al. (2013) found abnormal regional homogeneity (ReHo) values in the cerebellar lobes, highlighting altered local synchronization of neural activity in these regions [63]. These findings collectively suggest that Parkinson’s Disease involves widespread changes in brain network connectivity and regional efficiency, particularly affecting areas involved in motor control and coordination. These results suggest a progressive alteration in DMN connectivity correlating with the severity and type of Parkinson’s Disease symptoms. The increase in connectivity from CTL to ODN to ODP indicates a possible link between DMN dysfunction and the cognitive and sensory impairments associated with Parkinson’s Disease with hyposmia. The increase in brain functional connectivity observed in the Default Mode Network (DMN) in our study is consistent with findings reported by Yoneyama et al. 2018 and Mohammadi et al. (2012) [13,64]. Their research observed reduced connectivity in the left postcentral area and increased connectivity in the basal ganglia in Parkinson’s Disease patients. These changes suggest reorganizing functional networks, potentially as a compensatory mechanism to maintain cognitive and motor functions despite the neurodegenerative process. Our study has shown that connectivity within the cingulo-opercular network is disrupted in Parkinson’s Disease patients, contributing to deficits in cognitive and executive functions. This finding is consistent with Yoneyama et al. 2018, who have reported reduced network connectivity in the executive network [12]. The further reduction in connectivity observed in the ODP group compared to the ODN group highlights the potential impact of hyposmia on brain network connectivity. The distinct patterns of cingulo-opercular network connectivity among the groups underscore the potential of network connectivity measures as biomarkers for disease progression and subtype differentiation in Parkinson’s Disease.

### 4.6. Intervention

The findings from our study also have significant implications for developing targeted interventions for olfactory dysfunction in Parkinson’s Disease. Given that increased DMN connectivity correlates with the severity of hyposmia, interventions aimed at modulating DMN activity, such as repetitive transcranial magnetic stimulation (rTMS), could potentially mitigate olfactory deficits. Previous studies have shown that rTMS can improve motor symptoms and cognitive functions in Parkinson’s Disease patients by modulating brain network activity [65]. While rTMS has not shown significant effects on olfactory discrimination [66], its impact on other cognitive and motor symptoms suggests a need to explore its potential in addressing olfactory dysfunction. Moreover, understanding the therapeutic mechanisms of rTMS, such as its role in decreasing oxidative stress and increasing cerebral blood flow, could guide the optimization of rTMS protocols for targeting specific neural networks involved in olfactory processing. Improved intervention strategies could enhance the quality of life for Parkinson’s Disease patients by addressing both motor and non-motor symptoms, including olfactory dysfunction.

## 5. Conclusions

This research underscores the potential of graph theory to investigate altered connectivity in Parkinson’s Disease (PD) patients and its subtype. Despite minor discrepancies between PD studies due to differences in field strength and modality, the initial findings validate graph theory as an effective tool for probing connectivity changes in PD patients with normal cognitive ability, those with severe hyposmia, and healthy controls. In our study, the topology of the brain network of each group has been analyzed using graph theory. The rsfMRI data undergo pre-processing and are parcellated with the help of the Dosenbach-160 atlas. The connectivity matrix of the three groups has been estimated by employing Pearson correlation. The overall brain network topology helps in computing graph parameters. Statistical analysis was performed using the sparsity thresholding method and integrated network measurement method. The feature ranking has been used to identify the best graph theory metric to differentiate the groups. Our results have also suggested that the network topologically becomes altered in patients with Parkinson’s Disease. Brain network segregation, local efficiency, and small-worldness are reduced in Parkinson’s with normal cognitive ability (ODN) and are heavily worsened in Parkinson’s with severe hyposmia (ODP), both at the global and local levels. The network efficiency in the olfactory areas of limbic cortices, ventral middle prefrontal cortex, inferior parietal and occipital regions, and the cerebellum were severely damaged in ODP. The increased nodal clustering coefficient is noticed in the Default Mode Network of ODP compared to ODN as a compensatory effect. The present study has confirmed olfactory dysfunction and compensatory effects involved in the ODP group and hence serves as a supportive indicator for early Parkinson’s diagnosis. The resulting data will aid in the development of biomarkers for understanding the pathophysiological networks and cognitive dysfunctions in ODN and ODP through resting-state fMRI network modeling. This comprehensive assessment has the potential to deepen our understanding of the underlying pathophysiology of these diseases and influence the development of new therapeutic approaches focused on network modulation such as rTMS or tDCS.

## Figures and Tables

**Table 1:** Significant brain regions computed from nodal brain segregation (i.e., clustering coefficients) for (1) ODN vs. ODP (negative t-value indicates ODP has lower brain segregation), (2) HCs vs. ODP (negative t-value indicates ODP has lower brain segregation), and (3) HCs vs. ODN (negative t-value indicates ODN has lower brain segregation).

(1) Significant Brain Regions of ODN vs. ODP						
x-Corr	y-Corr	z-Corr	Brain Region	Network	*p*-Values	t-Values
−37	−54	−37	Left inferior cerebellum	cerebellum	0.0043	−3.1
−34	−67	−29	Left inferior cerebellum	cerebellum	0.0070	−2.9
−6	−79	−33	Left inferior cerebellum	cerebellum	0.0026	−3.3
18	−81	−33	Right inferior cerebellum	cerebellum	0.0019	−3.4
5	−75	−11	Right medial cerebellum	cerebellum	0.0052	−3.0
−36	18	2	Left anterior insula	cingulo-opercular	0.0008	−3.7
27	49	26	Right aPFC	cingulo-opercular	0.0005	−3.9
14	6	7	Right basal ganglia	cingulo-opercular	0.0011	−3.7
9	20	34	Right dACC	cingulo-opercular	0.0067	−2.9
0	15	45	mFC	cingulo-opercular	0.0030	−3.3
37	−2	−3	Right mid insula	cingulo-opercular	0.0048	−3.1
−55	−44	30	Left parietal	cingulo-opercular	0.0070	−2.9
42	−46	21	Right sup temporal	cingulo-opercular	0.0043	−3.1
−48	6	1	Left vFC	cingulo-opercular	0.0007	−3.8
5	−50	33	precuneus	default	0.0068	−2.9
−16	29	54	Left superior frontal	default	0.0005	−3.9
−6	50	−1	Left vmPFC	default	0.0060	−3.0
−11	45	17	Left vmPFC	default	0.0049	−3.1
44	8	34	Right dFC	fronto-parietal	0.0018	−3.4
−48	−47	49	Left IPL	fronto-parietal	0.0013	−2.9
36	−60	−8	Right occipital	occipital	0.0030	−3.3
39	−71	13	Right occipital	occipital	0.0020	−3.4
41	−23	55	Right parietal	sensorimotor	0.0040	−3.1
59	−13	8	Right temporal	sensorimotor	0.0049	−3.1
**(2) Significant Brain Regions of HCs vs. ODP**						
−25	−60	−34	Left inferior cerebellum	cerebellum	0.0004	−4.0
−34	−67	−29	Left inferior cerebellum	cerebellum	0.0002	−4.2
18	−81	−33	Right inferior cerebellum	cerebellum	0.0018	−3.5
21	−64	−22	Right lateral cerebellum	cerebellum	0.0034	−3.2
14	−75	−21	Right medial cerebellum	cerebellum	0.0035	−3.2
−2	30	27	ACC	cingulo-opercular	0.0011	−3.7
38	21	−1	Right anterior insula	cingulo-opercular	0.0004	−4.1
−36	18	2	Left anterior insula	cingulo-opercular	0.0001	−4.5
27	49	26	Right aPFC	cingulo-opercular	0.0075	−2.9
9	20	34	Right dACC	cingulo-opercular	0.0124	−2.7
37	−2	−3	Right middle insula	cingulo-opercular	0.0094	−2.8
58	−41	20	Right parietal	cingulo-opercular	0.0004	−4.0
8	−40	50	Right precuneus	cingulo-opercular	0.0005	−4.0
43	−43	8	Right temporal	cingulo-opercular	0.0081	−2.9
−12	−3	13	Left thalamus	cingulo-opercular	0.0002	−4.4
51	23	8	Right vFC	cingulo-opercular	0.0024	−3.3
−9	−72	41	Left occipital	default	0.0072	−2.9
−42	−76	26	Left occipital	default	0.0007	−3.8
−16	29	54	Left sup frontal	default	0.0008	−3.8
−1	28	40	ACC	fronto-parietal	0.0070	−2.9
40	17	40	Right dFC	fronto-parietal	0.0075	−2.9
44	8	34	Right dFC	fronto-parietal	0.0012	−3.6
54	−44	43	Right IPL	fronto-parietal	0.0067	−2.9
−48	−47	49	Left IPL	fronto-parietal	0.0100	−2.8
−34	−60	−5	Left occipital	occipital	0.0002	−4.3
39	−71	13	Right occipital	occipital	0.0104	−2.7
29	−73	29	Right occipital	occipital	0.0024	−3.3
−29	−75	28	Left occipital	occipital	0.0119	−2.7
53	−3	32	Right frontal	sensorimotor	0.0091	−2.8
−42	−3	11	Left middle insula	sensorimotor	0.0116	−2.7
−47	−12	36	Left parietal	sensorimotor	0.0092	−2.8
−47	−18	50	Left parietal	sensorimotor	0.0086	−2.8
41	−23	55	Right parietal	sensorimotor	0.0002	−4.3
18	−27	62	Right parietal	sensorimotor	0.0016	−3.5
−24	−30	64	Left parietal	sensorimotor	0.0121	−2.7
10	5	51	Right pre-SMA	sensorimotor	0.0002	−4.3
−44	−6	49	Left precentral gyrus	sensorimotor	0.0068	−2.9
59	−13	8	Right temporal	sensorimotor	0.0003	−4.1
−54	−22	9	Left temporal	sensorimotor	0.0013	−3.6
−53	−37	13	Left temporal	sensorimotor	0.0000	−5.7
43	1	12	Right vFC	sensorimotor	0.0003	−4.1
**(3) Significant Brain Regions of HCs vs. ODN**						
54	−44	43	Right IPL	fronto-parietal	0.0034	−3.2
−35	−46	48	Left post parietal	fronto-parietal	0.0022	−3.4

## Data Availability

The data that support the findings of this study are openly available in the OpenfMRI database at https://openneuro.org/datasets/ds000245/versions/00001 (accessed on 11 February 2020), accession number ds000245.

## Supplementary Materials

The following supporting information can be downloaded at: https://www.mdpi.com/article/10.3390/brainsci14070685/s1, Figure S1: Progressive decrease in olfactory region connectivity across groups. The figure illustrates brain connectivity changes in the olfactory regions obtained using CONN brain connectivity software. (a) Healthy controls exhibit the highest connectivity in the olfactory regions. (b) Individuals with Parkinson’s disease and normal cognition show a moderate decrease in connectivity. (c) Patients with Parkinson’s disease and severe hyposmia display the most significant reduction in olfactory region connectivity. This progressive decline highlights the impact of Parkinson’s disease on olfactory networks; Figure S2: Validation of dataset variability using deep neural network classification. The classification was performed to distinguish between healthy controls and Parkinson’s disease subjects with normal cognition. The training set consisted of 12 subjects from each group, while the validation set included 3 subjects from each group. The results demonstrate the accuracy of the deep neural network in classifying subjects separately, validating the variability within the dataset; Figure S3: Validation of dataset variability using deep neural network classification. The classification was performed to distinguish between Parkinson’s disease subjects with severe hyposmia and Parkinson’s disease subjects with normal cognition. The training set consisted of 12 subjects from each group, while the validation set included 3 subjects from each group. The results demonstrate the accuracy of the deep neural network in classifying subjects separately, validating the variability within the dataset.
