# Supplementary figures and images for: Disrupted Brain Network Measures in Parkinson’s Disease Patients with Severe Hyposmia and Cognitively Normal Ability
Source: Brain Sci. 2024 Jul 8;14(7):685. doi: 10.3390/brainsci14070685 (PMC11274763; doi:10.3390/brainsci14070685)

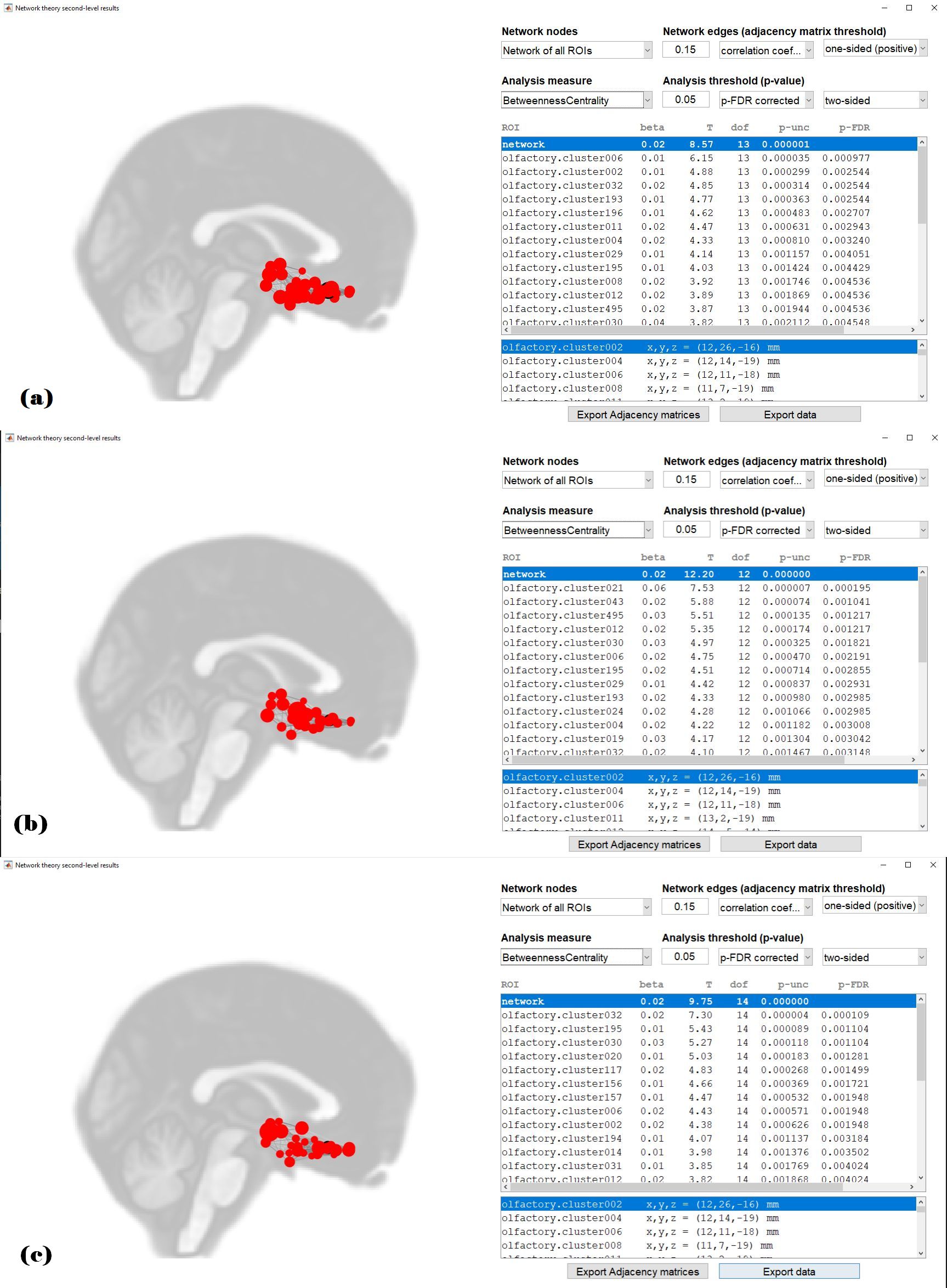

Supplement: Supplementary file 1 [file brainsci-14-00685-s001.zip › supplementary Result/Sub Fig 1/sub fig1.jpg]

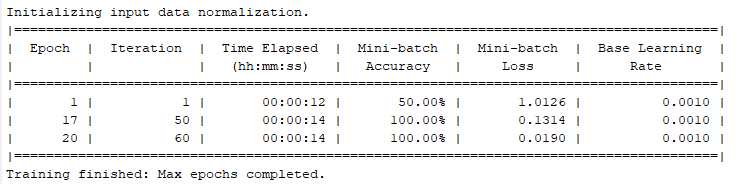

Supplement: Supplementary file 1 [file brainsci-14-00685-s001.zip › supplementary Result/Sub Fig2/11GT_N_C_acc.png]

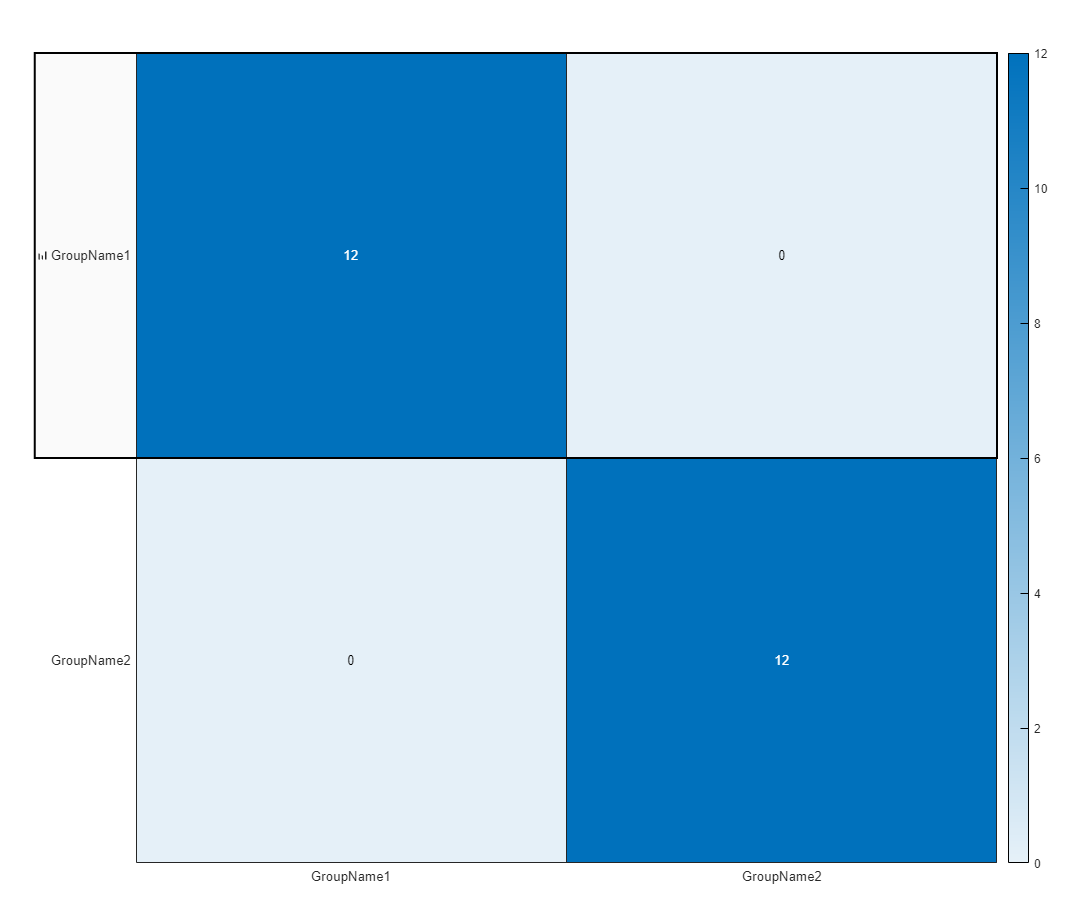

Supplement: Supplementary file 1 [file brainsci-14-00685-s001.zip › supplementary Result/Sub Fig2/11GT_N_C_cm.png]

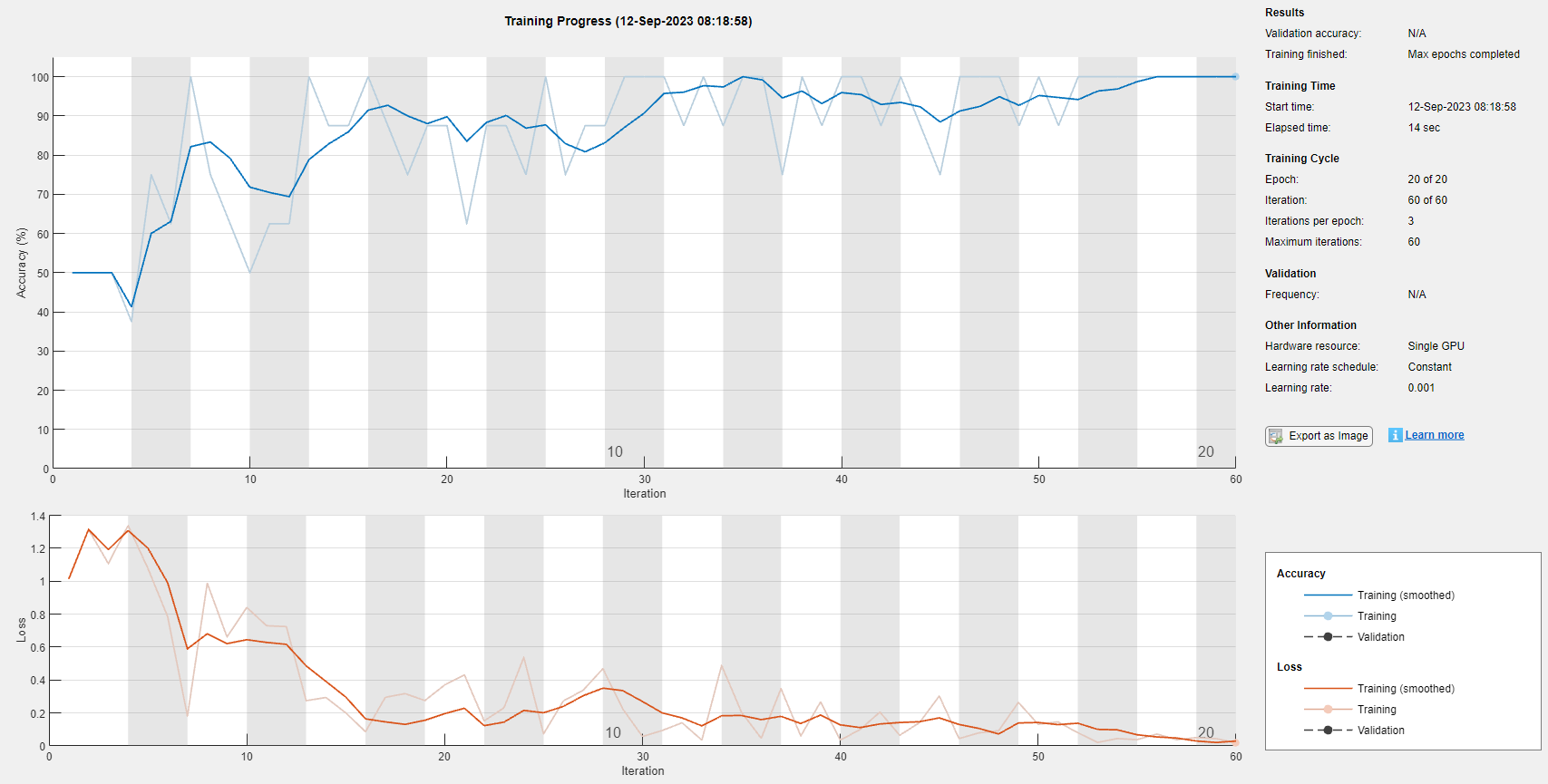

Supplement: Supplementary file 1 [file brainsci-14-00685-s001.zip › supplementary Result/Sub Fig2/11GT_N_C_train.png]

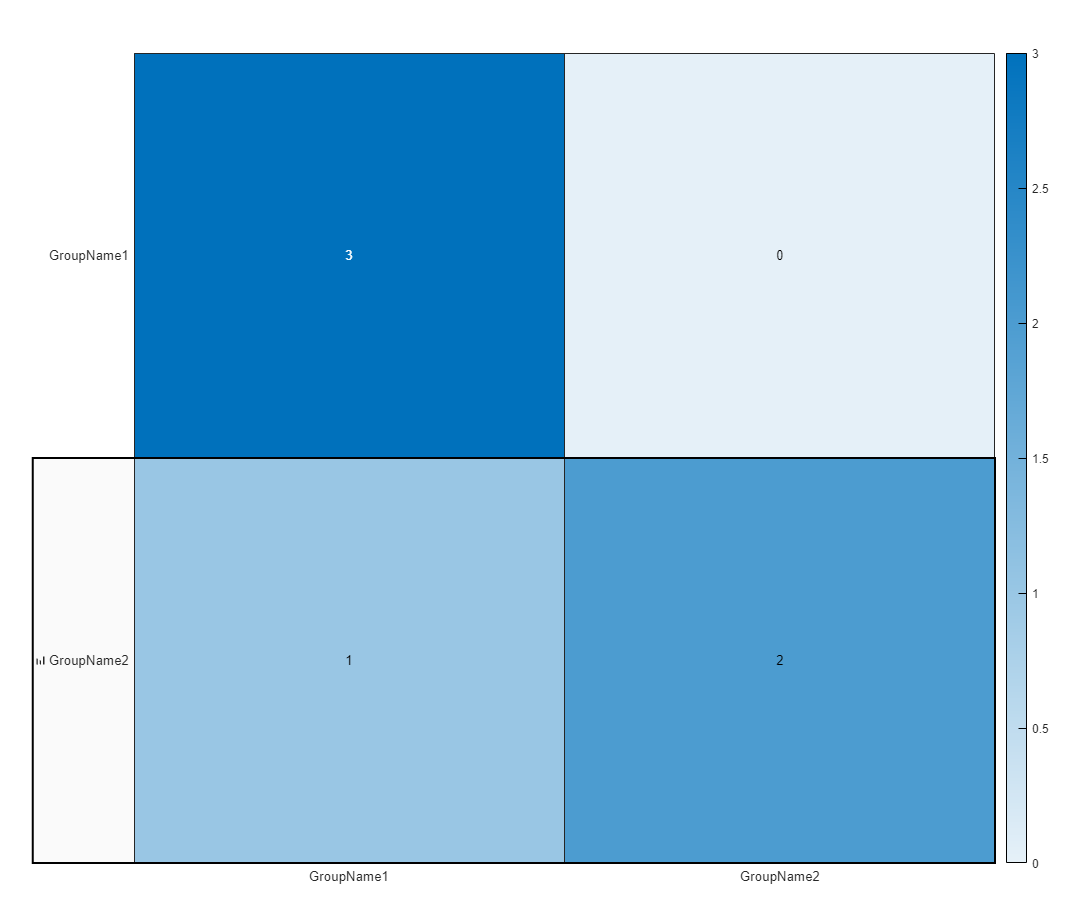

Supplement: Supplementary file 1 [file brainsci-14-00685-s001.zip › supplementary Result/Sub Fig2/11GT_N_C_valset cm.png]

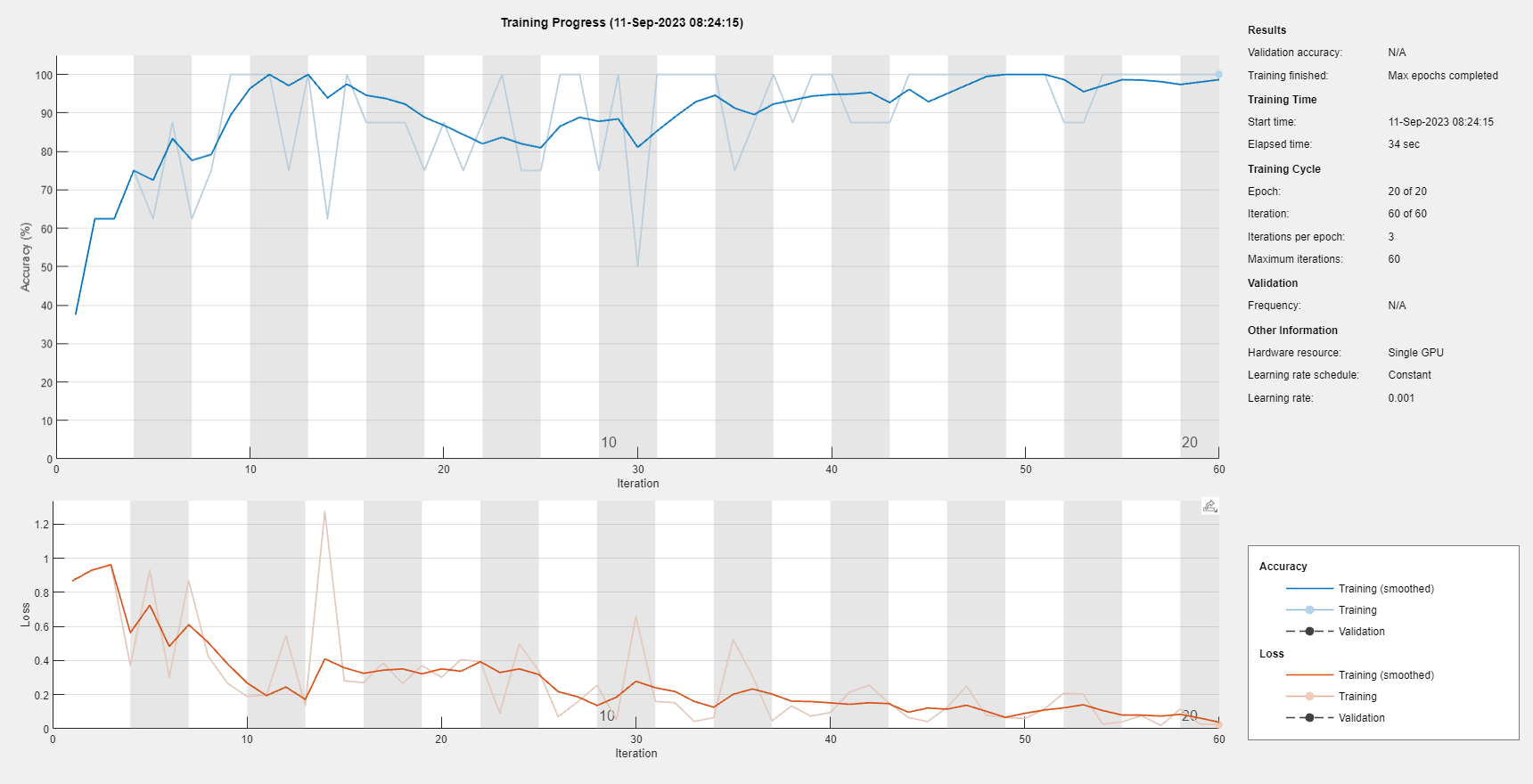

Supplement: Supplementary file 1 [file brainsci-14-00685-s001.zip › supplementary Result/Sub Fig3/11GT_P_N_train.png]

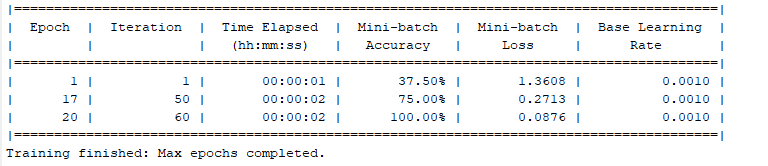

Supplement: Supplementary file 1 [file brainsci-14-00685-s001.zip › supplementary Result/Sub Fig3/11_GT_P_N_acc_valset.png]

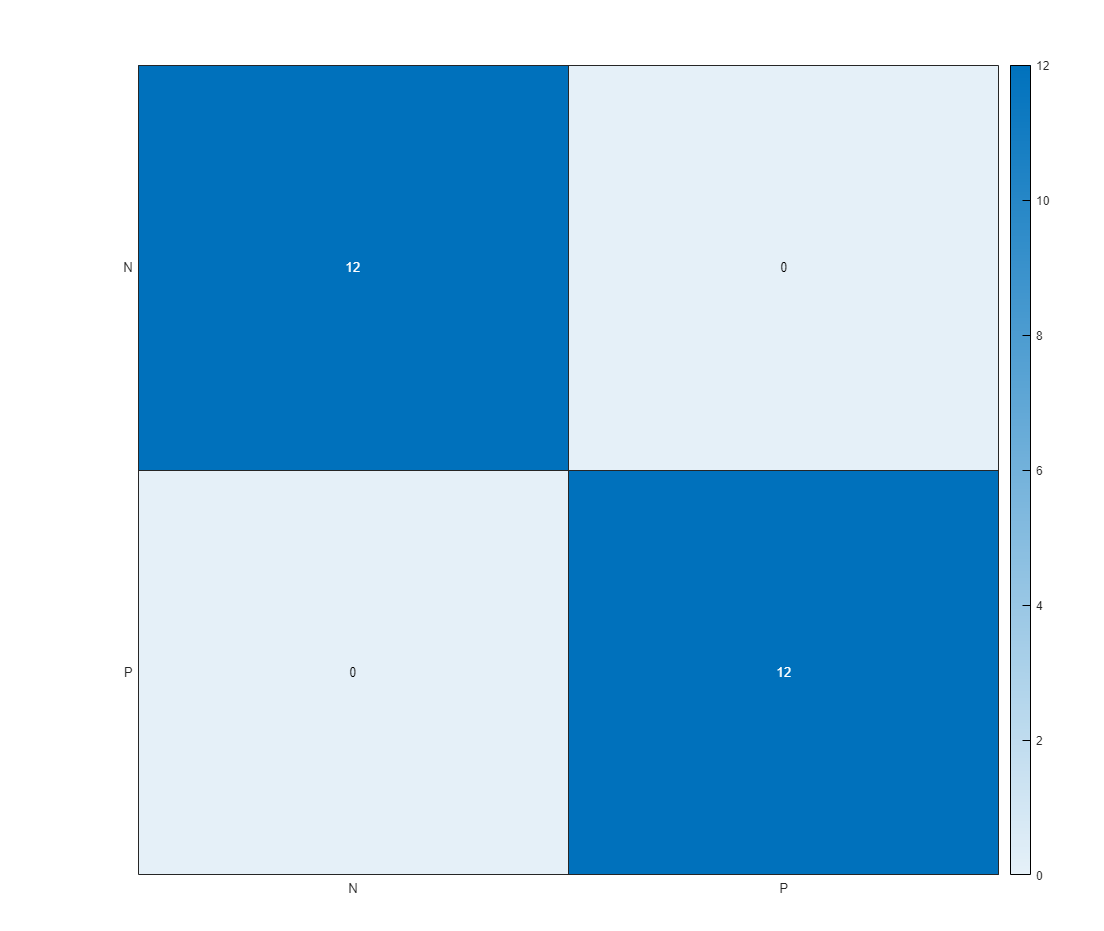

Supplement: Supplementary file 1 [file brainsci-14-00685-s001.zip › supplementary Result/Sub Fig3/11_GT_P_N_cm.png]

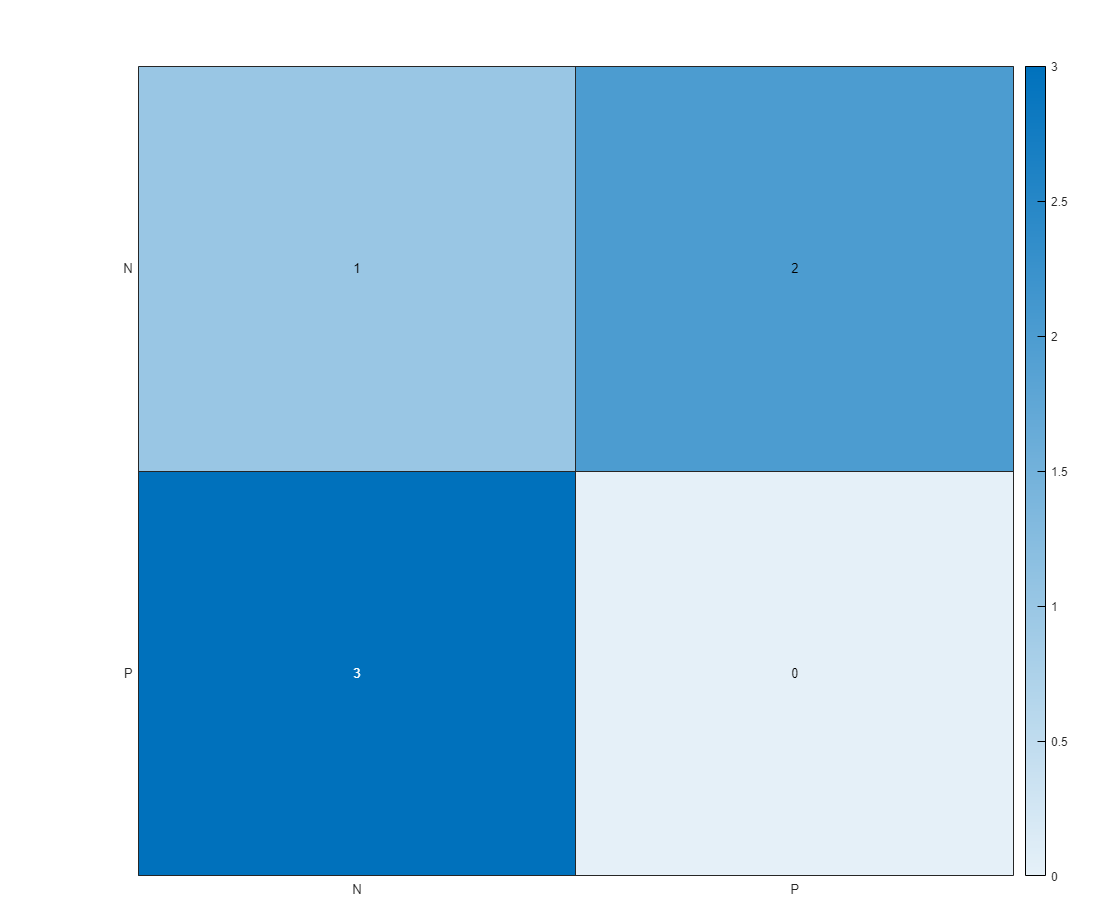

Supplement: Supplementary file 1 [file brainsci-14-00685-s001.zip › supplementary Result/Sub Fig3/11_GT_P_N_cm_valset.png]

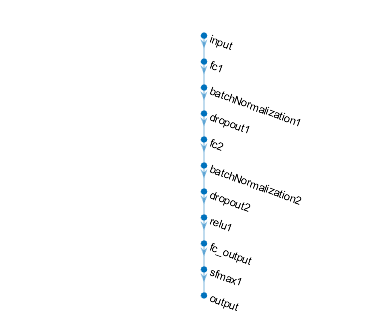

Supplement: Supplementary file 1 [file brainsci-14-00685-s001.zip › supplementary Result/Sub Fig3/Screenshot 2023-08-07 182925.png]
